# Global Profiling of Various Metabolites in *Platycodon grandiflorum* by UPLC-QTOF/MS

**DOI:** 10.3390/ijms161125993

**Published:** 2015-11-09

**Authors:** Jae Won Lee, Seung-Heon Ji, Geum-Soog Kim, Kyung-Sik Song, Yurry Um, Ok Tae Kim, Yi Lee, Chang Pyo Hong, Dong-Ho Shin, Chang-Kug Kim, Seung-Eun Lee, Young-Sup Ahn, Dae-Young Lee

**Affiliations:** 1 Department of Herbal Crop Research, National Institute of Horticultural and Herbal Science, RDA, Eumseong 369-873, Korea; jaewon3@gmail.com (J.W.L.); jiddung205@chungbuk.ac.kr (S.-H.J.); kimgs0725@korea.kr (G.-S.K.); urspower@korea.kr (Y.U.); kimot@korea.kr (O.T.K.); herbin3@korea.kr (S.-E.L.); ay21cay@korea.kr (Y.-S.A.); 2 College of Pharmacy and Research Institute of Pharmaceutical Sciences, Kyungpook National University, Daegu 702-701, Korea; kssong@knu.ac.kr; 3 Department of Industrial Plant Science and Technology, Chungbuk National University, Cheongju 361-763, Korea; leeyi22@cbnu.ac.kr; 4 Theragen Etex, Suwon 443-270, Korea; changpyo.hong@therabio.kr; 5 Genomics Division, National Academy of Agricultural Science (NAAS), Jeonju 560-500, Korea; dhshin013@korea.kr (D.-H.S.); chang@korea.kr (C.-K.K.)

**Keywords:** *Platycodon grandiflorum*, metabolomics, UPLC-QTOF/MS

## Abstract

In this study, a method of metabolite profiling based on UPLC-QTOF/MS was developed to analyze *Platycodon grandiflorum*. In the optimal UPLC, various metabolites, including major platycosides, were separated well in 15 min. The metabolite extraction protocols were also optimized by selecting a solvent for use in the study, the ratio of solvent to sample and sonication time. This method was used to profile two different parts of *P. grandiflorum*, *i.e.*, the roots of *P. grandiflorum* (PR) and the stems and leaves of *P. grandiflorum* (PS), in the positive and negative ion modes. As a result, PR and PS showed qualitatively and quantitatively different metabolite profiles. Furthermore, their metabolite compositions differed according to individual plant samples. These results indicate that the UPLC-QTOF/MS-based profiling method is a good tool to analyze various metabolites in *P. grandiflorum.* This metabolomics approach can also be applied to evaluate the overall quality of *P. grandiflorum*, as well as to discriminate the cultivars for the medicinal plant industry.

## 1. Introduction

*Platycodon grandiflorum*, a perennial herb grown widely in Northeast Asia, contains triterpenoid saponins, carbohydrates, fibers, *etc.* [1,2]. *P. grandiflorum* has been used as a food material and a traditional medicine. *Platycodin radix*, the root of *P. grandiflorum*, has various benefits for health, and its biological usefulness has been reviewed [3]. Interest in platycodin saponins, the main components of *P. grandiflorum*, has increased recently due to their novel pharmacological activities, including anti-inflammatory, anti-oxidant, anti-lipidemic, anti-obesity, anti-cancer activities and their ability to improve insulin resistance [4,5,6,7,8,9,10,11]. Furthermore, in a previous study, various components were isolated from the flower of *P. grandiflorum*, and their biological activities were monitored [12]. Thus, it is critical to study not only *Platycodin radix*, but also different parts of *P. grandiflorum* in order to screen it for useful components.

Recently, metabolomics approaches have been used to assess the metabolite contents of individual plant species [13]. For plant metabolomics, well-constructed analytical platforms are necessary [14,15]. Nuclear magnetic resonance spectroscopy and gas chromatography or liquid chromatography (LC) coupled with mass spectrometry (MS) have been widely used to analyze plant metabolites [16,17,18,19,20]. In particular, metabolite profiling by LC/MS is applicable to phenotype and can discriminate individual plant species [21,22,23]. In this study, we constructed a profiling method based on LC/MS to analyze the metabolites of *P. grandiflorum*. Metabolite extraction protocols and LC/MS conditions were optimized to profile two different parts ((1) *P. grandiflorum* roots (PR) and (2) *P. grandiflorum* stems and leaves (PS)).

## 2. Results and Discussion

### 2.1. Construction of LC-MS Conditions to Profile Platycodon grandiflorum Metabolites

**Figure 1:**
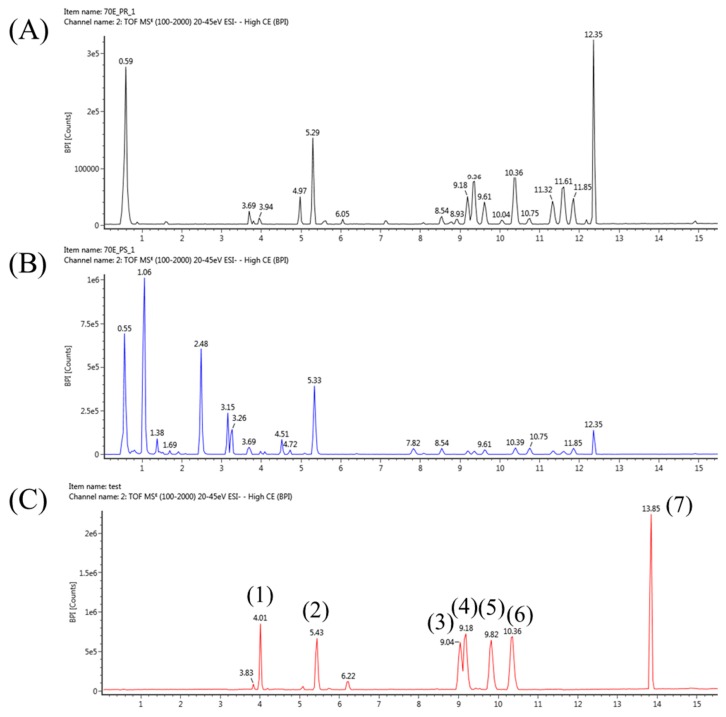
UPLC-QTOF/MS, ESI- base peak intensity (BPI) chromatogram of (**A**) *P. grandiflorum* roots; (**B**) *P. grandiflorum* stems and leaves and (**C**) seven standards, including (1) platycoside E, (2) platycodin D3, (3) platycodin D2, (4) platycodin D, (5) polygalacin D, (6) platycogenic acid A and (7) platycodigenin.

For the high-throughput and sensitive analysis of various metabolites in *P. grandiflorum*, it is necessary to construct a robust method of profiling. The UPLC system with its small particle size column enables the fast and effective separation of various molecules. Furthermore, QTOF/MS is a good tool for a full mass scan with high resolution. Thus, in this study, UPLC-QTOF/MS was applied to profile *P. grandiflorum* metabolites. First, we used seven standards (*i.e.*, platycoside E, platycodin D3, platycodin D2, platycodin D, polygalacin D, platycogenic acid A and platycodigenin) to optimize the LC-MS conditions. In the negative mode of electrospray ionization (ESI), seven compounds were mainly detected as [M − H]^−^ and [M + COOH]^−^ ions (Table 1). These standards were separated well and eluted for 15 min (Figure 1) at a flow rate of 450 µL/min by using an ACQUITY BEH C18 column (2.1 mm × 100 mm, 1.7 µm particle size). Second, two extracts of PR and PS were analyzed in both positive and negative ion modes. As a result, chromatographic data from the positive mode showed poor efficiency of ionization (data not shown). Thus, we carried out the profiling of PR and PS in the negative mode only. Various metabolites of PR and PS were also separated well in 15 min (Figure 1). The gradient elution program consisted of a first linear gradient from steady (A/B: 90/10) to solvent (A/B: 80/20) for 3 min; followed by a second linear gradient to solvent (A/B: 77/23) for 8 min; a third linear gradient to solvent (A/B: 5/95) for 9 min; and a forth linear gradient to solvent (A/B: 90/10) for 1 min. The column was equilibrated at 10% Solvent B for 4 min before reuse. The total run time was 25 min for each analysis. Third, we validated the performance of the *P. grandiflorum* metabolites’ profiling method. For this, we drew the standard curves of seven standards and calculated their linearity range and correlation. The limits of detection (LODs) of each isolated compound are also listed (Table 2).

**Table 1:** List of seven standards analyzed by UPLC-QTOF/MS. RT, retention time.

No.	Standards	RT (min)	Molecular Formula	Expected Neutral Mass (Da)	Observed Neutral Mass (Da)	QTOF/MS (ESI-) *m/z*	Mass Accuracy (ppm)	Adducts
1	Platycoside E	4.01	C_69_H_112_O_38_	1548.6832	1548.6783	1593.6765	−3.08	+HCOO
2	Platycodin D3	5.43	C_63_H_102_O_33_	1386.6303	1386.6268	1431.625	−2.44	+HCOO
3	Platycodin D2	9.04	C_63_H_102_O_33_	1386.6303	1386.6264	1385.6191	−2.84	-H
4	Platycodin D	9.18	C_57_H_92_O_28_	1224.5775	1224.5765	1269.5747	−0.82	+HCOO
5	Polygalacin D	9.82	C_57_H_92_O_27_	1208.5826	1208.5795	1253.5777	−2.5	+HCOO
6	Platycogenic acid A	10.36	C_57_H_90_O_29_	1238.5568	1238.5562	1237.5489	−0.5	-H
7	Platycodigenin	13.85	C_30_H_48_O_7_	520.34	520.3399	519.3326	−0.21	-H

**Table 2:** The linearity range, correlation (*R^2^*) and LOD of seven standards by UPLC-QTOF/MS.

No.	Standards	Correlation (*R*^2^)	Linear Range (ng)	LOD (ng)
1	Platycoside E	0.9939	1–40	1
2	Platycodin D3	0.9975	1–100	1
3	Platycodin D2	0.9976	1–100	1
4	Platycodin D	0.9898	1–40	1
5	Polygalacin D	0.9926	0.2–100	0.2
6	Platycogenic acid A	0.9957	1–100	1
7	Platycodigenin	0.9952	0.2–20	0.2

### 2.2. Optimization of Extraction Protocols for Platycodon grandiflorum Metabolites

Next, we optimized the protocols to extract metabolites, including various isolated compounds from two different parts (PR and PS). There are several factors involved in the optimization of extraction protocols. First, it is critical to select suitable solvents for the extraction of various metabolites with different polarities. A single solvent is insufficient to dissolve the wide range of compounds, and several solvents, such as water, ethanol (EtOH) and methanol (MeOH), have been widely used to extract plant metabolites. To find the most effective solvent for extraction, we tried to compare six different solvents (50% EtOH, 70% EtOH, 80% EtOH, 50% MeOH, 70% MeOH and 80% MeOH) while other factors, such as solvent amount (20 mL) and sonication time (30 min), were kept constant. As a result, 70% EtOH exhibited the largest number of peaks and the highest intensity of several compounds. Second, the ratio of solvent to sample is also a critical factor in the metabolites’ extraction due to the limited solubility of samples. Thus, we compared four different amounts (10, 20, 30 and 40 mL) of solvent, 70% EtOH, to extract metabolites from the 50 mg of samples while the sonication time (30 min) was kept constant. Among them, 40 mL provided the largest amount of extracts. Third, different sonication times (15, 30, 45, 60 and 75 min) were tested in the use of 40 mL of 70% EtOH, and as a result, it was found that at least 60 min of sonication were required to extract many metabolites. Finally, we optimized the solvent used (70% EtOH), the ratio of solvent to sample (40 mL:50 mg) and the sonication time (60 min) for the effective extraction of metabolites from *P. grandiflorum*.

### 2.3. Analysis of Various Metabolites in the Stem, Leaf and Roots of *Platycodon grandiflorum*

**Figure 2:**
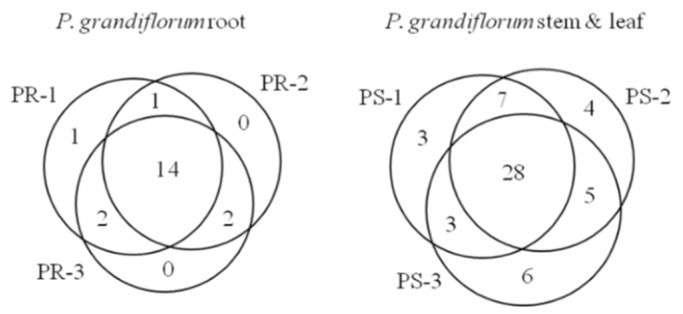
Venn diagram of metabolites analyzed in three *P. grandiflorum* roots (PRs) and three *P. grandiflorum* stems and leaves (PSs) (ESI-).

**Figure 3:**
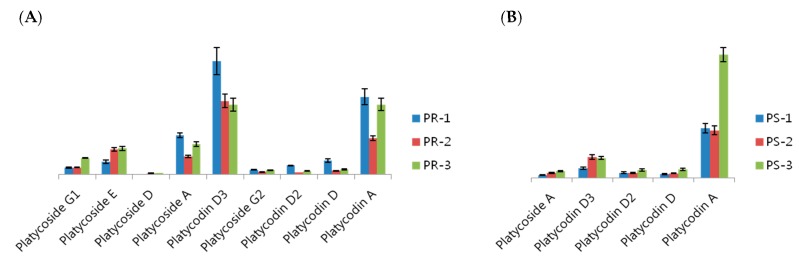
Bar graph of several platycosides in three PRs (**A**) and three PSs (**B**).

We applied the proposed method based on UPLC-QTOF/MS to profile various metabolites in PR and PS. Three species of *P. grandiflorum* were used to obtain three PRs and three PSs. We extracted the metabolites from the three PRs and three PSs, respectively, analyzed each extract three times (*n* = 3) and then processed each set of data using the UNIFI^TM^ software (Version 1.7.1; Waters Corp.). As a result, various metabolites, including several platycosides, were identified based on the library of UNIFI software containing information for the molecule’s name and formula [24]. From the processed data, compounds identified repeatedly (*n* = 3) having high mass accuracy (ppm < ±5) are listed in Table 3 and Table 4, respectively. In the peak assignment, it is critical to estimate retention time (RT) (RT tolerance: 0.2 min) and mass accuracy. In the samples of PR and PS, four compounds, such as platycoside E, platycodin D, platycodin D2 and platycodin D3, were analyzed with small RT shifts compared to the standard analysis. We also describe the Venn diagram of metabolites analyzed in three PRs and three PSs to show how the metabolic compositions of each plant species (PR-1, -2 and -3 or PS-1, -2 and -3) were different (Figure 2). Two parts of *P. grandiflorum* (PR and PS) showed qualitatively and quantitatively different metabolite profiles. Furthermore, the metabolite profiles differed according to individual samples. In particular, several platycosides were abundantly different in the three PRs and three PSs, respectively (Figure 3). For example, comparing to other PR species, PR-1 has 1.5-times more abundant platycodin D3, and PR-2 has two-times less abundant platycodin A. Compared to other PS species, PS-3 also has 2.5-times more abundant platycodin A.

**Table 3:** List of metabolites analyzed in *P. grandiflorum* root (ESI-).

No.	RT (min)	Metabolites	Expected Neutral Mass (Da)	Observed Neutral Mass (Da)	QTOF/MS (ESI-) *m/z*	Mass Accuracy (ppm)	Adducts	Intensity (*n* = 3)		
								PR-1	PR-2	PR-3
1	0.55	Glutamine	146.0691	146.0688	145.0615	−2.5	-H	^a^ 1837 ± 187	-	-
2	0.55	Planteose	504.169	504.169	549.1672	0	+HCOO	4903 ± 667	-	5636 ± 392
3	0.55	α-Kojibiose	342.1162	342.1161	387.1143	−0.22	-H	1837 ± 958	16,344 ± 1310	-
4	0.6	Maleic acid	116.011	116.0113	115.004	2.98	-H	-	487 ± 66	483 ± 31
5	0.62	2-Hydroxyfuran-3-formic acid	128.011	128.0104	173.0086	−3.5	+HCOO	2305 ± 106	2025 ± 113	1752 ± 44
6	0.63	2-Furoic acid	112.016	112.0163	111.009	1.99	-H	7646 ± 355	6875 ± 226	5943 ± 171
7	0.63	Citric acid	192.027	192.0264	191.0191	−3.21	-H	41,368 ± 2527	37,122 ± 1795	32,419 ± 1422
8	2.11	Arnebifuranone	316.1311	316.1310	361.1292	−0.19	+HCOO	661 ± 20	889 ± 28	656 ± 31
9	2.35	Brusatol	520.1945	520.1934	565.1917	−1.8	+HCOO	4005 ± 351	2836 ± 266	5753 ± 492
10	3.8	Platycoside G1	1416.6409	1416.6364	1461.6346	−3.11	+HCOO	2456 ± 195	2457 ± 100	5734 ± 106
11	3.97	Platycoside E	1548.6832	1548.6788	1593.677	−2.74	+HCOO	4451 ± 655	8863 ± 674	9183 ± 699
12	3.99	Lobetyolin	396.1784	396.1785	441.1767	0.19	+HCOO	13,407 ± 265	5473 ± 271	6794 ± 393
13	4.15	Platycoside D	1532.6882	1532.679	1577.6772	−5.83	+HCOO	-	385 ± 47	500 ± 30
14	4.98	Platycoside A	1254.5881	1254.5849	1299.5831	−2.48	+HCOO	13,802 ± 805	6425 ± 435	10,745 ± 864
15	5.31	Platycodin D3	1386.6303	1386.6261	1431.6243	−2.96	+HCOO	40,086 ± 4797	25,927 ± 2368	24,630 ± 2328
16	5.67	Platycoside G2	1284.5986	1284.5937	1283.5865	−3.82	-H	1626 ± 140	926 ± 103	1503 ± 149
17	8.24	Deapioplatycodin D	1092.5353	1092.5333	1137.5315	−1.74	+HCOO	658 ± 47	-	443 ± 45
18	8.8	Platycodin D2	1386.6303	1386.6231	1385.6159	−5.2	-H	3130 ± 171	744 ± 44	1172 ± 103
19	8.93	Platycodin D	1224.5775	1224.5717	1269.5699	−4.58	+HCOO	^a^ 4868 ± 606	1255 ± 67	1757 ± 161
20	9.35	Platycodin A	1266.5881	1266.5844	1311.5826	−2.82	+HCOO	27,391 ± 2780	12,819 ± 889	24,750 ± 2250

^a^ The values are the mean of intensities ±SD (*n* = 3).

**Table 4:** List of metabolites analyzed in *P. grandiflorum* stems and leaves (ESI-).

No.	RT (min)	Metabolites	Expected Neutral Mass (Da)	Observed Neutral Mass (Da)	QTOF/MS (ESI-) *m/z*	Mass Accuracy (ppm)	Adducts	Intensity (*n* = 3)		
								PS-1	PS-2	PS-3
1	0.51	Arginine	174.1117	174.1114	173.1041	−1.65	-H	^a^ 477 ± 38	594 ± 37	-
2	0.55	Aspartic acid	133.0375	133.0374	132.0301	−0.79	-H	405 ± 53	-	-
3	0.55	Glutamic acid	147.0532	147.0529	146.0456	−1.59	-H	464 ± 47	506 ± 30	-
4	0.55	Glutamine	146.0691	146.0692	145.0619	0.14	-H	1306 ± 223	2516 ± 304	-
5	0.55	Planteose	504.169	504.1687	549.1669	−0.57	+HCOO	619 ± 59	-	-
6	0.55	Sodium ferulate	216.0399	216.0391	215.0318	−3.58	-H	996 ± 77	-	924 ± 36
7	0.55	α-Kojibiose	342.1162	342.1162	387.1144	−0.03	+HCOO	-	-	10,644 ± 1655
8	0.56	Ribose	150.0528	150.0522	195.0504	−3.26	+HCOO	1321 ± 83	1488 ± 103	1994 ± 138
9	0.6	Maleic acid	116.011	116.0113	115.004	2.99	-H	2127 ± 103	2421 ± 125	1743 ± 58
10	0.61	5-Oxoproline	129.0426	129.0427	128.0354	0.88	-H	-	643 ± 49	-
11	0.61	Pyruvic acid	88.016	88.0161	133.0143	0.3	+HCOO	10,969 ± 482	11,961 ± 423	8726 ± 469
12	0.62	2-Furoic acid	112.016	112.0164	111.0091	3.1	-H	4606 ± 239	5134 ± 222	3194 ± 252
13	0.62	2-Hydroxyfuran-3-formic acid	128.011	128.0106	173.0088	−2.23	+HCOO	^a^ 1611 ± 92	1806 ± 111	1397 ± 60
14	0.62	Citric acid	192.027	192.0263	191.019	−3.61	-H	26,784 ± 677	30,360 ± 1294	18,681 ± 375
15	0.77	6,7-Dihydroxycoumarin	178.0266	178.0261	223.0243	−2.23	+HCOO	1090 ± 83	677 ± 33	513 ± 27
16	0.8	1-*O*-Caffeoyquinic acid	354.0951	354.0951	353.0878	0.05	-H	-	-	4272 ± 498
17	1	Decaffeoylacteoside	462.1737	462.1741	461.1669	0.91	-H	339 ± 46	494 ± 50	329 ± 33
18	1.01	Tryptophan	204.0899	204.0899	203.0826	−0.01	-H	813 ± 83	974 ± 106	566 ± 34
19	1.06	Quinic acid	192.0634	192.063	191.0557	−2.21	-H	26,641 ± 1875	27,777 ± 2173	31,286 ± 1747
20	1.07	4-*O*-Caffeoylquinic acid	354.0951	354.0951	353.0878	−0.08	-H	-	-	83,405 ± 3981
21	1.1	Shikimic acid	174.0528	174.0527	173.0454	−0.78	-H	591 ± 50	624 ± 77	811 ± 90
22	1.15	1,5-Dihydrxy-2,3,4,7-tetramethoxyxanthone	348.0845	348.0838	393.082	−1.77	+HCOO	354 ± 20	-	329 ± 25
23	1.15	4-*O*-β-d Glucopyranosyl-cis-cinnamic acid	326.1002	326.1002	325.0929	0.08	-H	1445 ± 147	-	1392 ± 86
24	1.21	Genistein-7,4'-di-*O*-β-d-glucoside	594.1585	594.1583	593.151	−0.32	-H	-	1280 ± 176	-
25	1.26	1-*_O_*-Caffeoyl-β-d-glucopyranoside	342.0951	342.095	341.0878	−0.14	-H	2243 ± 143	2603 ± 289	2525 ± 148
26	1.35	*o*-Coumaric acid	164.0473	164.0472	163.04	−0.6	-H	670 ± 118	859 ± 177	962 ± 102
27	1.38	Chlorogenic acid	354.0951	354.095	353.0877	−0.27	-H	12,530 ± 967	14,301 ± 1076	14,725 ± 798
28	1.91	3-*O*-trans-Coumaroylquinic acid	338.1002	338.1001	337.0928	−0.24	-H	3879 ± 282	6685 ± 483	3502 ± 257
29	2.04	9-Hydroxylinalool-9-β-d-glucopyranoside	332.1835	332.1834	377.1816	−0.35	+HCOO	680 ± 116	431 ± 77	868 ± 111
30	2.11	Arnebifuranone	316.1311	316.131	361.1292	−0.1	+HCOO	-	373 ± 32	-
31	2.24	Kaempferol-3-*O*-neohesperidoside	594.1585	594.1581	593.1508	−0.69	-H	-	-	^a^ 1578 ± 158
32	2.37	Brusatol	520.1945	520.1944	565.1926	−0.08	+HCOO	-	6136 ± 483	4755 ± 461
33	2.4	Kaempferol-3-gentiobioside	610.1534	610.1523	609.145	−1.86	-H	-	1030 ± 284	-
34	2.49	Kaempferol	286.0477	286.0467	285.0394	−3.62	-H	-	-	4400 ± 366
35	2.5	Quercetin-3-*O*-glucuronide 6″-methyl ester	492.0904	492.0907	491.0834	0.58	-H	1028 ± 88	900 ± 100	-
36	2.53	Taraxacolide 1-glucopyranoside	430.2203	430.2204	475.2186	0.3	+HCOO	3078 ± 242	4529 ± 519	3659 ± 430
37	3	1,5-*O*-Dicaffeoylquinic acid	516.1268	516.1269	515.1196	0.25	-H	-	-	510 ± 31
38	3.27	3'-Hydroxypuerarin	448.1006	448.1009	447.0936	0.75	-H	1894 ± 208	2107 ± 231	2228 ± 243
39	3.27	Eriodictyol-7-*O*-β-d-methyl-glucuronopyranoside	478.1111	478.111	477.1038	−0.19	-H	-	750 ± 67	975 ± 61
40	3.37	Luteolin 7-*O*-(6''- *O*-acetyl)-β-d-glucopyranoside	490.1111	490.1112	489.1039	0.13	-H	790 ± 58	21,846 ± 1788	7148 ± 472
41	3.37	Quercetin-3-*O*-(6"-*O*-acetyl)-β-d-glucopyranoside	506.106	506.107	505.0997	1.91	-H	-	369 ± 34	763 ± 116
42	3.38	Epicatechin-3-*O*-gallate	442.09	442.0907	487.0889	1.49	+HCOO	-	3838 ± 400	1207 ± 95
43	3.58	5-*O*-Caffeoyl quinic acid butyl ester	410.1577	410.1579	409.1506	0.5	-H	328 ± 28	516 ± 96	361 ± 21
44	3.62	Kaempferol-3-*O*-(6′′-*O*-acetyl)-β-d-glucopyranoside	490.1111	490.1122	489.1049	2.14	-H	74,782 ± 6202	-	-
45	3.99	Methyl-β-d-fructofuranoside	180.0634	180.0632	179.0559	−0.9	-H	1944 ± 224	2168 ± 222	-
46	4.98	Platycoside A	1254.588	1254.585	1299.583	−2.28	+HCOO	417 ± 48	815 ± 147	1088 ± 108
47	5.11	5,7,3′,5′-Tetrahydroxyflavanone	288.0634	288.0629	287.0556	−1.81	-H	3719 ± 239	2445 ± 142	-
48	5.24	Epigallocatechin	594.1373	594.137	593.1298	−0.49	-H	-	-	581 ± 78
49	5.31	Platycodin D3	1386.63	1386.625	1431.623	−3.83	+HCOO	^a^ 1582 ± 209	3569 ± 375	3396 ± 291
50	5.35	5,7,2',5'-Tetrahydroxy-flavone	286.0477	286.0473	285.04	−1.48	-H	152,282 ± 7073	146,318 ± 5227	136,503 ± 4826
51	5.36	5,7,8,3′,4′-Pentamethoxy flavones	302.0427	302.0424	301.0352	−0.71	-H	542 ± 63	558 ± 76	515 ± 45
52	6.27	Apigenin-7-*O*-β-d-glucuronide ethyl ester	474.1162	474.1166	473.1093	0.79	-H	2009 ± 109	1265 ± 145	-
53	7.83	Apigenol	270.0528	270.0522	269.0449	−2.32	-H	43,135 ± 2315	59,953 ± 3044	38,068 ± 1691
54	8.8	Platycodin D2	1386.63	1386.626	1385.618	−3.45	-H	849 ± 156	792 ± 113	1307 ±205
55	8.94	Platycodin D	1224.578	1224.574	1269.572	−2.86	+HCOO	613 ± 88	763 ± 87	1416 ± 190
56	9.37	Platycodin A	1266.588	1266.584	1311.583	−2.91	+HCOO	8564 ± 832	8149 ± 769	21,255 ± 1202

^a^ The values are the mean of intensities ±SD (*n* = 3).

## 3. Experimental Section

### 3.1. Platycodon grandiflorum Samples

The samples of *Platycodon grandiflorum* (roots, stems and leaves) were purchased from a Daegu Herbal Market in Daegu Gyeongbuk Province, Korea, in 2014. Voucher specimen (NIHHS150128) was deposited at the herbarium of the Department of Herbal Crop Research, National Institute of Horticultural and Herbal Science, Rural Development Administration, Eumseong, Korea.

### 3.2. Standard Constituents and Reagents

HPLC-grade acetonitrile, methanol and water were obtained from Merck (Darmstadt, Germany). Formic acid was purchased from Sigma-Aldrich (St. Louis, MO, USA). Standard compounds were isolated and purified from *Platycodon grandiflorum* roots by a series of chromatography procedures in our laboratory, and their structures were elucidated by a comparison of the spectroscopic data (MS, ^1^H-NMR and ^13^C-NMR) with the literature data: platycoside E [25], platycodin D3 [26], platycodin D [27], platycodin D2 [26], polygalacin D [26], platycogenic acid A [2] and platycodigenin [28]. The purity of the isolated compounds was determined to be more than 98% by normalization of the peak areas detected by HPLC analysis.

### 3.3. Sample Preparation

Each sample was dried at 40 °C in a forced-air convection-drying oven for 48 h after washing, and then weighed. The main and lateral roots were used in experiments after removing the rhizomes and fine roots. The roots were ground (<0.5 mm) using a mixer (Hanil, Seoul, Korea) and thoroughly mixed, after which the subsamples were homogenized further using a Retsch MM400 mixer mill (Retsch GmbH, Haan, Germany) for the analyses. Fine powder was weighed (50 mg), suspended in 40 mL of 70% (*v*/*v*) ethanol and ultrasonically extracted for 1 h at 50 °C. The extract was filtered and evaporated by using a solvent evaporator, Genevac Ez-2 (Genevac Ltd, Suffolk, UK), and the residue (5 mg) was dissolved in the 1 mL of 70% methanol. The solution was filtered through a syringe filter (0.22 µm) and injected directly into the UPLC system.

### 3.4. UPLC-QTOF-MS Analysis

UPLC was performed using a Waters ACQUITY H-Class UPLC (Waters Corp, Milford, MA, USA). Chromatographic separations were performed on an ACQUITY BEH C18 column (2.1 mm × 100 mm, 1.7 µm). The column oven was maintained at 40 °C, and the mobile phases consisted of Solvent A (5% acetonitrile + 0.1% formic acid (*v*/*v*)) and Solvent B (95% acetonitrile + 0.1% formic acid (*v*/*v*)). The flow rate was 450 μL/min, and the injection volume was 2 μL for each run. Next, MS analysis was performed using a Waters Xevo G2-S QTOF MS (Waters Corp.) operating in positive and negative ion mode. The mass spectrometers performed alternative high- and low-energy scans, known as the MS^E^ acquisition mode. The operating parameters were set as follows: cone voltage, 40 V; capillary, 3.0 kV; source temperature, 120 °C; desolvation temperature, 300 °C; cone gas flow, 30 L/h; and desolvation gas flow, 600 L/h. Accurate mass measurements were obtained by means of an automated calibration delivery system, which contains the internal reference (Leucine, *m/z* 556.276 (ESI+), *m/z* 554.262(ESI-)). Data were collected between 100 and 2000 *m/z*. In the quantitative analysis of each metabolite, the height of peaks was used to measure the intensity.

## 4. Conclusions

A profiling method based on UPLC-QTOF/MS was developed to analyze various metabolites contained in *P. grandiflorum*. UPLC separation conditions were optimized by using seven isolated compounds. The protocols for extracting *P. grandiflorum* metabolites were optimized as follows: solvent used, 70% EtOH; the ratio of solvent to sample, 40 mL:50 mg; and sonication time, 60 min. We applied this method to profile two different parts of *P. grandiflorum* (PR and PS), in what was a first attempt to characterize various metabolites in PR and PS, respectively. In the negative ion modes, PR and PS showed qualitatively and quantitatively different metabolite profiles. The metabolite compositions also differed according to the each species. These results indicated that the UPLC-QTOF/MS-based profiling method has potential as a tool to analyze various metabolites in *P. grandiflorum*. Hence, this study is of great significance regarding evaluations of the overall quality of *P. grandiflorum* in pharmacological and clinical investigations of drug products. Furthermore, this metabolomics approach could be applied to discriminate cultivars for use in the agricultural and pharmacological industries.

## References

[1] Tada A., Kaneiwa Y., Shoji J., Shibata S. (1975). Studies on the saponins of the root of *Platycodon grandiflorum* A. DE CANDOLLE. I. Isolation and the structure of platycodin-D. Chem. Pharm. Bull..

[2] Choi Y.H., Yoo D.S., Choi C.W., Cha M.R., Kim Y.S., Lee H.S., Lee K.R., Ryu S.Y. (2008). Platyconic Acid A, a Genuine Triterpenoid Saponin from the Roots of *Platycodon grandiflorum*. Molecules.

[3] Nyakudya E., Jeong J.H., Lee N.K., Jeong Y.S. (2014). Platycosides from the Roots of *Platycodon grandiflorum* and Their Health Benefits. Prev. Nutr. Food Sci..

[4] Chung J.W., Noh E.J., Zhao H.L., Sim J.S., Ha Y.W., Shin E.M., Lee E.B., Cheong C.S., Kim Y.S. (2008). Anti-inflammatory Activity of Prosapogenin Methyl Ester of Platycodin D via Nuclear Factor-kappaB Pathway Inhibition. Biol. Pharm. Bull..

[5] Zhao H.L., Cho K.H., Ha Y.W., Jeong T.S., Lee W.S., Kim Y.S. (2006). Cholesterol-lowering effect of platycodin D in hypercholesterolemic ICR mice. Eur. J. Pharmacol..

[6] Lee J.Y., Hwang W.I., Lim S.T. (2004). Antioxidant and anticancer activities of organic extracts from *Platycodon grandiflorum* A. De Candolle roots. J. Ethnopharmacol..

[7] Jeong C.H., Choi G.N., Kim J.H., Kwak J.H., Kim D.O., Kim Y.J., Heo H.J. (2010). Antioxidant activities from the aerial parts of *Platycodon grandiflorum*. Food Chem..

[8] Noh J.R., Kim Y.H., Gang G.T., Yang K.J., Kim S.K., Ryu S.Y., Kim Y.S., Lee C.H., Lee H.S. (2010). Preventative Effects of *Platycodon grandiflorum* Treatment on Hepatic Steatosis in High Fat Diet-Fed C57BL/6 Mice. Biol. Pharm. Bull..

[9] Kim K.S., Ezaki O., Ikemoto S., Itakura H. (1995). Effects of *Platycodon Grandilorum* Feeding on Serum and Liver Lipid Concentrations in Rats with Diet-Induced Hyperlipidemia. J. Nutr. Sci. Vitaminol..

[10] Ravipati A.S., Zhang L., Koyyalamudi S.R., Jeong S.C., Reddy N., Bartlett J., Smith P.T., Shanmugam K., Münch G., Wu M.J. (2012). Antioxidant and anti-inflammatory activities of selected Chinese medicinal plants and their relation with antioxidant content. BMC Complem. Altern. Med..

[11] Kim K.S., Seo E.K., Lee Y.C., Lee T.K., Cho Y.W., Ezaki O., Kim C.H. (2000). Effect of dietary *Platycodon grandiflorum* on the improvement of insulin resistance in obese Zucker rats. J. Nutr. Biochem..

[12] Jang D.S., Lee Y.M., Jeong I.H., Kim J.S. (2010). Constituents of the Flowers of *Platycodon grandiflorum* with Inhibitory Activity on Advanced Glycation End Products and Rat Lens Aldose Reductase *In Vitro*. Arch. Pharm. Res..

[13] Schauer N., Fernie A.R. (2006). Plant metabolomics: towards biological function and mechanism. Trends Plant. Sci..

[14] Sumner L.W., Mendes P., Dixon R.A. (2003). Plant metabolomics: Large-scale phytochemistry in the functional genomics era. Phytochemistry..

[15] Fukusaki E., Kobayashi A. (2005). Plant Metabolomics: Potential for Practical Operation. J. Biosci. Bioeng..

[16] Ward J.L., Baker J.M., Beale M.H. (2007). Recent applications of NMR spectroscopy in plant metabolomics. FEBS J..

[17] Krishnan P., Kruger N.J., Ratcliffe R.G. (2005). Metabolite fingerprinting and profiling in plants using NMR. J. Exp. Bot..

[18] t’Kindt R., Morreel K., Deforce D., Boerjan W., Bocxlaer J.V. (2009). Joint GC–MS and LC–MS platforms for comprehensive plant metabolomics: Repeatability and sample pre-treatment. J. Chromatogr. B.

[19] Grata E., Boccard J., Guillarme D., Glauser G., Carrupt P.A., Farmer E.E., Wolfender J.L., Rudaz S. (2008). UPLC–TOF-MS for plant metabolomics: A sequential approach for wound marker analysis in *Arabidopsis thaliana*. J. Chromatogr. B.

[20] Steinmann D., Ganzera M. (2011). Recent advances on HPLC/MS in medicinal plant analysis. J. Pharmaceut. Biomed..

[21] Vos R.C.D., Moco S., Lommen A., Keurentjes J.J., Bino R.J., Hall R.D. (2007). Untargeted large-scale plant metabolomics using liquid chromatography coupled to mass spectrometry. Nat. Protoc..

[22] Kim N., Choi B.Y., Lee D., Shin Y.S., Bang K.H., Cha S.W., Lee J.W., Choi H.K., Jang D.S., Lee D. (2011). Metabolomic Approach for Age Discrimination of *Panax ginseng* Using UPLC-Q-Tof MS. J. Agric. Food Chem..

[23] Xie G.X., Ni Y., Su M.M., Zhang Y.Y., Zhao A.H., Gao X.F., Liu Z., Xiao P.G., Jia W. (2008). Application of ultra-performance LC-TOF MS metabolite profiling techniques to the analysis of medicinal Panax herbs. Metabolomics.

[24] Natural Products Application Solution with UNIFI：Waters. http://www.waters.com/waters/en_US/Natural-Products-Application-Solution-withUNIFI/nav.htm?cid=134777097&lset=1&locale=en_US&changedCountry=Y.

[25] He Z., Qiao C., Han Q., Wang Y., Ye W., Xu H. (2005). New triterpenoid saponins from the roots of *Platycodon grandiflorum*. Tetrahedron.

[26] Ishii H., Tori K., Tozyo T., Yoshimura Y. (1984). Saponins from roots of *Platycodon grandiflorum*. Part 2. Isolation and structure of new triterpene glycosides. J. Chem. Soc. Perkin Trans. 1.

[27] Li W., Xiang L., Zhang J., Zheng Y.N., Han L.K., Saito M. (2007). A new triterpenoid saponin from the roots of *Platycodon. grandiflorum*. Chin. Chem. Lett..

[28] Choi Y.H., Yoo D.S., Cha M.R., Choi C.W., Kim Y.S., Choi S.U., Lee K.R., Ryu S.Y. (2010). Antiproliferative effects of saponins from the roots of *Platycodon. grandiflorum* on cultured human tumor cells. J. Nat. Prod..

